# Retracted: Activation of CD137 Signaling Enhances Vascular Calcification through c-Jun N-Terminal Kinase-Dependent Disruption of Autophagic Flux

**DOI:** 10.1155/2022/9856896

**Published:** 2022-04-06

**Authors:** Mediators of Inflammation

*Mediators of Inflammation* has retracted the article titled “Activation of CD137 Signaling Enhances Vascular Calcification through c-Jun N-Terminal Kinase-Dependent Disruption of Autophagic Flux” [1] because of image overlap with the authors previously published work. Specifically:

- Figure 1(a) [1] Von Kossa panels appear identical to Figure 1 [2]
- Figure 1(a) [1] LC3B, Beclin 1 and p62 panels appear identical to Figure 2 [2]
- Figure 1(e) [1] appears identical to Figure 7 [2]
- Figure 3(e) [1] appears identical to Figure 5 [2]
- Figure 5(d) [1] appears identical to Figure 6 [3]

The authors were unresponsive to communications regarding this retraction.
